# Population-Based Pertussis Incidence and Risk Factors in Infants Less Than 6 Months in Nepal

**DOI:** 10.1093/jpids/piw079

**Published:** 2017-01-10

**Authors:** Michelle M Hughes, Janet A Englund, Jane Kuypers, James M Tielsch, Subarna K Khatry, Laxman Shrestha, Steven C LeClerq, Mark Steinhoff, Joanne Katz

**Affiliations:** 1 Johns Hopkins Bloomberg School of Public Health, Department of International Health, Global Disease Epidemiology and Control, Baltimore, Maryland; 2 University of Washington, Seattle Children’s Hospital, Seattle; 3 University of Washington, Molecular Virology Laboratory, Seattle; 4 George Washington University Milken Institute School of Public Health, Department of Global Health, Washington, District of Columbia; 5 Nepal Nutrition Intervention Project–Sarlahi, Kathmandu; 6 Tribhuvan University Teaching Hospital, Department of Paediatrics, Institute of Medicine, Maharajgunj, Kathmandu, Nepal; 7 Cincinnati Children’s Hospital and Medical Center, Global Health Center, Ohio

**Keywords:** epidemiology, incidence, infants, Nepal, pertussis

## Abstract

**Background:**

Pertussis is estimated to cause 2 percent of childhood deaths globally and is a growing public health problem in developed countries despite high vaccination coverage. Infants are at greatest risk of morbidity and mortality. Maternal vaccination during pregnancy may be effective to prevent pertussis in young infants, but population-based estimates of disease burden in infants are lacking, particularly in low-income countries. The objective of this study was to estimate the incidence of pertussis in infants less than 6 months of age in Sarlahi District, Nepal.

**Methods:**

Nested within a population-based randomized controlled trial of influenza vaccination during pregnancy, infants were visited weekly from birth through 6 months to assess respiratory illness in the prior week. If any respiratory symptoms had occurred, a nasal swab was collected and tested with a multitarget pertussis polymerase chain reaction (PCR) assay. The prospective cohort study includes infants observed between May 2011 and August 2014.

**Results:**

The incidence of PCR-confirmed *Bordetella pertussis* was 13.3 cases per 1000 infant-years (95% confidence interval, 7.7–21.3) in a cohort of 3483 infants with at least 1 day of follow-up.

**Conclusions:**

In a population-based active home surveillance for respiratory illness, a low risk for pertussis was estimated among infants in rural Nepal. Nepal’s immunization program, which includes a childhood whole cell pertussis vaccine, may be effective in controlling pertussis in infants.

A resurgence of pertussis across age groups has occurred in several countries in recent years [1]. Middle- and high-income countries that use an acellular pertussis vaccine for the primary vaccination series have been particularly affected [2, 3], and infants and adolescents have experienced the greatest increase [4]. Factors that may contribute to the increased risk of pertussis include rapidly waning immunity from those vaccinated with acellular vaccines [1, 5, 6], asymptomatic transmission from individuals vaccinated with acellular vaccines [7], genetic adaption of *Bordetella pertussis* [8], vaccination delay or refusal [9], improved surveillance and laboratory capabilities [2], and overall increased awareness of the continuing circulation of *B pertussis* [1].

Some countries experiencing epidemic pertussis, including the United States, United Kingdom, and Argentina, now recommend pertussis immunization in pregnancy and vaccination of close contacts [10, 11] to protect the youngest infants from pertussis before they can be vaccinated themselves [12]. Recent data from maternal vaccination trials demonstrate the ability of antibodies to be transferred from mothers to their infants in pregnancy and their persistence in infants [13].

Global estimates of pertussis show the highest childhood burden in Southeast Asia [14]. In this region, maternal pertussis vaccination during pregnancy may be a way to protect infants, similar to the approach using tetanus toxoid vaccine. However, globally only 1 population-based estimate of pertussis in infants from birth has been conducted (Senegal) [15], and surveillance and laboratory capabilities in Asia are lacking [16, 17]. The World Health Organization (WHO) recently recommended that countries using whole cell pertussis vaccines continue to do so in light of recent data indicating that acellular pertussis vaccines are less effective than whole cell pertussis vaccines [18]. Population-based data are needed, especially in low-income settings, to provide a more accurate estimate of the burden of pertussis in infants to inform childhood and maternal immunization policies [19, 20].

We report on a prospective cohort study following infants weekly in their homes to monitor for pertussis disease from birth to age 6 months. The objective was to provide a population-based estimate of laboratory-confirmed pertussis incidence in infants less than 6 months of age in the Sarlahi District, Nepal.

## METHODS

### Settings and Population

The study was nested within 2 consecutive randomized controlled trials of maternal influenza vaccination during pregnancy set in the Sarlahi District, located in the central Terai (low-lying plains) region of Nepal [21]. At the start of the trial, prevalent pregnancies were identified through a census of all households in the catchment area. For the duration of the trial, field workers visited all households in the communities, every 5 weeks, where married women (15–40 years) resided, for surveillance of incident pregnancies. Once a pregnancy was identified, women provided consent and were enrolled. From April 25, 2011 through September 9, 2013, women between 17 and 34 weeks gestation were randomized and vaccinated with either an influenza vaccine or placebo. The study was a population-based prospective cohort of infants followed from birth through 6 months postpartum. Approval for the study was obtained from the Institutional Review Boards at the Johns Hopkins Bloomberg School of Public Health, Cincinnati Children’s Medical Center, the Institute of Medicine at Tribhuvan University, Kathmandu, and the Nepal Health Research Council. The trials are registered at Clinicaltrials.gov (NCT01034254).

### Data Collection

At baseline, information was collected on household structure, socioeconomic status, and demographics. At enrollment, date of last menstrual period and pregnancy history data were collected. As soon as possible after delivery, the mother and infant were visited to collect detailed birth information including infant weight and breastfeeding status. From birth through 6 months, postpartum infants were visited weekly by a field worker, who recorded any infant respiratory symptoms in the past 7 days. If an infant had any of the following symptoms, a mid-nasal nylon flocked swab was collected: fever, cough, wheeze, difficulty breathing, or ear infection. Starting on August 17, 2012, new symptoms, more specific for pertussis, were added to the weekly morbidity visit: apnea, cyanosis, cough with vomit, or whoop/whooping cough. The swabs were stored for up to 1 week at room temperature in PrimeStore Molecular Transport Medium (Longhorn Diagnostics LLC, Bethesda, MD). In addition to these signs, mothers were asked which, if any, infant vaccinations were received in the past 7 days, including pertussis vaccination [22]. Mid-nasal swabs were also collected on a weekly basis from mothers from enrollment through 6 months postpartum who reported fever plus one additional morbidity (cough, sore throat, nasal congestion, or myalgia). All nasal swabs collected from infants were tested for *B pertussis*, *Bordetella parapertussis*, and *Bordetella bronchispetica*. Only the nasal swabs of mothers whose infants tested positive for any of these pathogens were tested for the same pathogens.

### Laboratory Assays

Real-time polymerase chain reaction (PCR) testing was conducted at the University of Washington’s Molecular Virology Laboratory according to previously published methods [23]. Two-target PCR was used to assess the presence of 3 *Bordetella* species: *B pertussis*, *B parapertussis*, and *B bronchiseptica*. The amplified targets were chromosomal repeated insertion sequence IS481 (IS) and the polymorphic pertussis toxin ptxA promoter region (PT).

After amplification, the melting points of the amplicons were measured in an iCycler (Bio-Rad). A sample was interpreted as positive when the target(s) had a melting temperature within the species-specific acceptable range and a computed tomography ≤42. A sample was negative if none of the targets tested positive or a single positive target was not reproducible. Maternal nasal swabs were tested for those mothers whose infants tested positive for any *Bordetella* species

Polymerase chain reaction was also performed for several viral infections (influenza, rhinovirus [RV], respiratory syncytial virus [RSV], bocavirus [BoV], human metapneumovirus, coronavirus, adenovirus, and parainfluenza [1–4]) as previously described [21].

### Analytic Dataset

Of 3693 women enrolled, 3646 infants were live born to 3621 women (Supplementary Figure 1). Infants were included in this analysis if they were followed for any length of the follow-up period (0 to 180 days); median total follow-up was 146 days per infant (Supplementary Figure 2). The final dataset consists of 3483 infants, contributing 1280 infant-years of observation, with at least 1 follow-up visit during the first 6 months. This includes infants from the entire trial period, both before and after more pertussis-specific additions to the weekly symptom questionnaire.

At baseline, data on household structure were gathered. At enrollment, women reported their literacy status (binary) and pregnancy history. The field workers identified their ethnicity into 2 broad groups (Pahadi, a group originating from the hills; or Madeshi, a group originating from north India) from names and observation. Women were categorized as nulliparous or multiparous. Responses to 25 questions about household construction, water and sanitation, and household assets were used to develop an index to measure the socioeconomic status of households. Binary variables for each of the 25 questions and a mean SES score were calculated for each household.

Gestational age was measured using a woman’s report of date of last menstrual period during pregnancy surveillance. Birth weight was collected as soon as possible after birth using a digital scale (Tanita model BD-585, precision to nearest 10 grams). Birth weights collected >72 hours after birth were excluded from the analysis. Small for gestational age (SGA) was calculated using the sex-specific 10th percentile cutoff described by Alexander et al [24] and the INTERGROWTH-21 standards [25]. Women were asked within how many hours of birth breastfeeding was initiated and binary breastfeeding categories were created (≤1 hour versus >1 hour postdelivery).

### Statistical Analysis

Incidence was calculated as the number of pertussis cases per 1000 infant-years at risk. Poisson exact 95% confidence intervals (CIs) were constructed. Characteristics of infant pertussis cases were compared with nonpertussis cases using bivariate Poisson regression. Characteristics of all pertussis respiratory episodes were compared with nonpertussis respiratory episodes; *t* tests were used for continuous predictors and Fisher’s exact tests were used for categorical associations due to the low number of pertussis episodes. All statistical analyses were conducted in Stata/SE 14.1.

## RESULTS

A total of 3483 infants had 4283 episodes of respiratory illness between May 18, 2011 and April 30, 2014. Thirty-nine percent (n = 1350) of infants experienced no respiratory episodes. The incidence of respiratory illness was 3.6 episodes per infant-year (95% CI, 3.5–3.7). Mean episode duration was 4.7 days (95% CI, 4.6–4.9). A total of 3930 (92%) episodes were matched to 1 or more pertussis-tested nasal swabs from 2026 infants (Supplementary Figure 1).

Seventeen cases of *B pertussis* were identified from 19 nasal swabs (nasal swabs were positive on 2 consecutive weeks for 2 infants). The incidence of PCR-confirmed *B pertussis* was 13.3 cases per 1000-infant years (95% CI, 7.7–21.3). Five cases of *B parapertussis* were detected with an incidence of 3.9 cases per 1000 infant-years (95% CI, 1.3–9.1). No cases of *B bronchiseptica* were identified.

### Bordetella Pertussis

**Figure 1.**
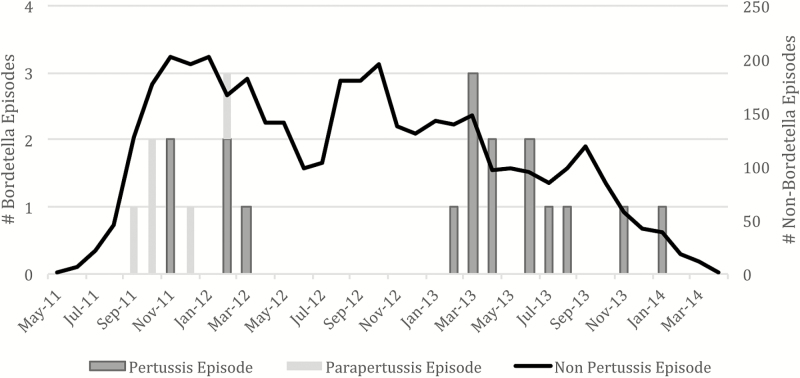
Timing of respiratory episodes.

The average pertussis episode duration was 8 days (range, 2–33) (Table 1). Mean age of onset of symptoms was 83 days (range, 19–137) (median, 80; interquartile range, 63–109). The most common symptoms were cough, difficulty breathing, and cough with vomit. None of the additional symptoms related to pertussis that were added in year 2 (cyanosis, apnea, cough with vomit, and whoop) resulted in collection of nasal swabs based solely on these additional symptoms. Pertussis episodes were statistically significantly more likely to include difficulty breathing, cough with vomit, and whoop compared with other respiratory illness. Six infants had at least 1 pertussis vaccination before pertussis disease onset (three <2 weeks and three >2 weeks before pertussis illness) with a mean of 18 days from vaccination to illness compared with 49 days for nonpertussis episodes (*P* = .03). Five infants received their first pertussis vaccination postpertussis disease onset, whereas 6 infants received no pertussis vaccination in the first 180 days. Three fourths of pertussis episodes were coinfected with at least 1 virus, with RV and BoV the most common. Cases of pertussis were more likely to be infected with BoV than respiratory cases due to causes other than pertussis. The majority of cases occurred between February 2013 and January 2014 (Figure 1).

**Table 1.** Comparison of Pertussis Episodes to Nonpertussis Episodes

	Nonpertussis Episodes		Pertussis Episodes		
	(n = 3913)		(n = 17)		*P* Value^a^
Characteristic	Proportion	Mean	Proportion	Mean	
Symptoms^b^					
Cough	62%		71%		.62
Difficulty breathing	40%		65%		**.05**
Cough with vomit	12%		50%		**.00**
Wheeze	45%		47%		.99
Fever	53%		47%		.64
Whoop	6%		33%		**.01**
Apnea	4%		17%		.08
Cyanosis	1%		8%		.09
Ear Infection	5%		6%		.59
Episode duration (days)		5		8	.07
Age at episode start (days)		91		83	.54
Coinfections					
RV	50%		53%		.99
BoV	5%		24%		**.01**
PIV3	4%		12%		.17
RSV	9%		6%		.99
Influenza	5%		6%		.55
MPV	5%		6%		.57
CoV	8%		6%		.99
PIV1	2%		0%		.99
PIV2	1%		0%		.99
PIV4	2%		0%		.99
AdV	2%		0%		.99
Vaccination					
Received 1st pertussis vaccination	38%		35%		0.99
Days since vaccination		49		18	**0.03**

Abbreviations: AdV, adenovirus; BoV, bocavirus; CoV, coronavirus; MPV, human metapneumovirus; PIV, parainfluenza; RSV, respiratory syncytial virus; RV, rhinovirus.

^a^ *t* tests were used for continuous predictors and Fisher’s exact tests were used for categorical predictors; statistical significance of P < .05 indicated in bold.

^b^Cough with vomit, apnea, whoop, and cyanosis were only captured in year 2; denominator for these symptoms was 2034 episodes.

No statistically significant differences between risk factors for pertussis and nonpertussis cases (Table 2) were documented. Given the low number of pertussis cases, the lack of a statistical association is not evidence of nonassociation. No deaths occurred in infants who had pertussis. Of the 8 mothers of *B pertussis*-positive infants who had a nasal swab collected (14 nasal swabs total) during their own follow-up, none were positive for any pertussis species.

**Table 2.** Poisson Regression for Risk Factors for Pertussis in Infants

Risk Factor	Nonpertussis Infants	Pertussis Infants	Unadjusted		
	(n = 3466)	(n = 17)	IRR	95% CI	*P* Value
Male Sex	53%	59%	1.3	0.5 − 3.4	.61
Preterm (<37 weeks)	12%	24%	2.2	0.7 − 6.6	.18
Low birth weight (<2500 grams)	25%	36%	1.7	0.5 − 5.9	.38
Small for gestational age (IG)	37%	50%	1.7	0.5 − 5.8	.41
Small for gestational age (A)	48%	55%	1.3	0.4 − 4.3	.65
Breastfed in 1st hour	35%	38%	1.1	0.4 − 3.0	.86
Primiparous	42%	59%	2.0	0.8 − 5.2	.16
Pahadi ethnicity	58%	69%	1.6	0.6 − 4.6	.37
Literate	61%	60%	1.6	0.6 − 4.6	.37
Household size (mean)	5	4	0.9	0.7 − 1.1	.39
SES score (mean)	0.39	0.35	0.2	0.0 − 8.6	.42
Age (days) at first pertussis vaccination (mean)	85	96	1.0	1.0 − 1.0	.17
1st pertussis vaccination received by 6 months	56%	65%	1.5	0.5 − 3.9	.47

Abbreviations: A, Alexander standards; CI, confidence interval; IG, INTERGROWTH-21st standards; IRR, incidence rate ratios; SES, socioeconomic status.

### Bordetella Parapertussis

The 5 *B parapertussis* cases were primarily male whose mothers were primiparous, literate, and Pahadi ethnicity (Supplementary Table 1). No mothers of infants who had *B parapertussis* had a nasal swab collected during follow-up.

The average *B parapertussis* episode duration was 4 days (Supplementary Table 2). Mean age of onset of symptoms was 58 days with a range of 7–95 days. The most common symptoms were cough and wheeze. Rhinovirus and RSV were the only coinfections observed. All *B parapertussis* cases occurred between September 2011 and February 2012 (Figure 1).

## DISCUSSION

A low incidence of pertussis and generally mild clinical presentation were found in infants <6 months in Nepal. To our knowledge, this represents one of the first population-based active surveillance of PCR-confirmed pertussis among young infants in Asia. Acellular pertussis vaccine trials conducted in the 1990s found the average pertussis incidence in the whole cell vaccine groups ranged from 1 to 37 cases per 1000 infant-years [26]. Our finding of 13 *B pertussis* cases per 1000 infant-years was on the lower end of this range. In the United States in 2014, the estimated pertussis incidence in infants less than 6 months was 2 cases per 1000 infant-years [27], much lower than observed in our study; however, this passive surveillance system likely vastly underestimates pertussis incidence. Thus, there is a need for active surveillance data such as ours. Furthermore, given our highly sensitive case detection method, many of our pertussis cases would likely not have been detected in the previous acellular pertussis vaccine trials. More stringent respiratory symptom criteria would have lowered our incidence estimate even further. The low incidence was found in a population where pentavalent vaccine (Pentavac: Diphtheria, Tetanus, Pertussis [Whole Cell], Hepatitis-B and Haemophilus Type b Conjugate Vaccine; Serum Institute of India Pvt. Ltd), scheduled for administration at 6, 10, and 14 weeks, is received with significant delays (7% of infants received all 3 recommended pertussis vaccines by 6 months) [22]. These data support the WHO’s recommendation that countries using whole cell pertussis vaccine continue to do so given that the majority of outbreaks have been concentrated in countries using the acellular pertussis vaccine [2]. Recent studies suggest that protection from acellular pertussis vaccine is not as strong or long lasting as that conferred by the whole cell pertussis vaccine [6, 28].

Another contributing factor to the low pertussis incidence observed could be that surveillance was conducted during a period of low pertussis transmission. Pertussis is a cyclical disease, thought to peak every 2 to 4 years, and we may have captured the burden at a low circulation period [6]. We observed over 70% of our *B pertussis* cases over a 1-year period. This increase from earlier observation periods could indicate a temporary rise in pertussis consistent with its cyclical pattern or a true increase in the baseline burden. Previous research on pertussis seasonality has in different places and time periods demonstrated various periods of peak transmission or no discernable patterns [29, 30]. Although our data do not support a seasonal pattern, the numbers observed are too low to be conclusive.

Pertussis symptom duration and severity were mild compared with the classic pertussis case presentation. Only 3 of the 17 cases fulfilled the WHO criteria, which requires a minimum of 2 weeks of cough, whoop, or posttussive vomiting [31]. Studies on pertussis in infants have generally been clinic-based, hospital-based, or in an outbreak, which therefore required a certain severity of illness for parents to recognize a need for medical attention [29, 30, 32]. These study designs and passive surveillance efforts therefore may have missed milder pertussis cases [33]. Our study, which required only 1 respiratory symptom for a nasal swab to be collected, had increased sensitivity to detect a range of pertussis case presentations. An alternative explanation for the mild cases seen could be an increase in the proportion of mild compared with severe pertussis cases in Nepal.

Although cough, difficulty breathing, and cough with vomit were the most common symptoms, no symptom was present in all *B pertussis* cases. During an epidemic period in Washington state, among infants <1 year, who had a minimum of 14 days cough plus an additional symptom, 82% had posttussive emesis, 29% had apnea, 26% had whoop, and 42% had cyanosis [32]. A study of US neonates with pertussis showed the symptom prevalence to be 97% for cough, 91% for cyanosis, 58% for apnea, and 3% for fever [34]. Our study found lower or equal symptom prevalence with the exception of fever. Fever prevalence was higher in our study, similar to that found in Peru [29].

Although not statistically significant, infants with pertussis were more likely to have been born preterm, low birth weight, and SGA, and their mothers were more likely to be primiparous. These findings are similar to previous studies showing no difference in pertussis cases by sex [29, 35, 36] or crowding [35] but showing differences by birth weight [36]. Coinfections were common, consistent with findings from other hospital-based studies [33]. Codetection of *B pertussis* and *B parapertussis* with respiratory viruses may be due to asymptomatic pertussis carriage.

The incidence of *B parapertussis* of 4 cases per 1000 person-years was comparable to that of 2 per 1000 person-years found in the Italian acellular pertussis vaccine trial in 1992–1993 [37]. The duration of illness was shorter for *B parapertussis* with a maximum duration of 6 days compared with a maximum of 33 days for *B pertussis*. A milder presentation is consistent with clinical knowledge of *B parapertussis* infection [37, 38]. *Bordetella parapertussis* cases occurred only during a 5-month period.

### Limitations

There were several study design limitations. We cannot be certain whether the reported symptoms were caused by pertussis, another organism, or whether symptoms were related to 2 or more etiologic agents. We were unable to perform multivariate regression modeling for characteristics associated with pertussis disease and pertussis cases due to the small number of cases we detected.

Infant respiratory symptoms were reported by parents, who may have missed signs that might have been observed by a healthcare worker. However, the criteria for collection of the nasal swab were broad and did not require sophisticated clinical skills. However, apnea and cyanosis may have been difficult for parents to identify. Although the criteria for specimen collection changed in year 2, no infant experienced a pertussis-specific symptom in isolation without also having one of the originally specified respiratory symptoms. These data support our assumption that we were unlikely to have missed pertussis cases in year 1 with our less sensitive respiratory symptom criteria.

Nasal swabs were collected in the mid-nasal region for influenza virus detection, which may have lowered the sensitivity of pertussis detection. In a field site, the acceptability of an additional nasopharyngeal swab would likely have increased the participant refusal rate. This would have decreased the generalizability of our results to the entire population. Although nasopharyngeal swabs or nasopharyngeal aspirates are the recommended specimen collection method [39], the nasopharyngeal region was established as the collection area of choice when the diagnostic measure was culture, which has low sensitivity. Recent data demonstrated the comparability of using mid-nasal versus nasopharyngeal swabs in PCR pertussis detection [40].

### Strengths

Strengths of the study included being a population-based, prospective study, with very low refusal rates. Risk factors, clinical symptoms, and coinfections were prospectively identified without the potential bias that may occur when these data are collected retrospectively or in clinical settings. The community-based design allows generalizability of these results to the entire population and not just those seeking care at a health facility or in an outbreak situation. The Sarlahi District is located in the Terai region where the majority of Nepalese reside, and it has similar demographics to the entire population of Nepal [41]. Sarlahi’s location near sea level and on the border with India supports the generalizability of these results to many populations living on the Indian subcontinent. The weekly active surveillance with sensitive criteria for pertussis testing was able to detect mild and atypical pertussis cases, which may have been missed by previous traditional surveillance. The multitarget PCR method allowed highly sensitive and specific detection of 2 additional *Bordetella* species beyond the primary *B pertussis* target.

## CONCLUSIONS

We observed a low incidence of pertussis in infants in a whole cell vaccine environment. Pertussis cases were generally milder than expected compared with traditional pertussis clinical definitions. These data support clinicians considering pertussis in their differential diagnosis of infants with mild respiratory symptoms. Policymakers in Nepal will need to weigh the benefit of an additional prenatal pertussis vaccine or a switch to acellular primary pertussis vaccine with the low burden of pertussis in infants less than 6 months.

Our study demonstrated that mid-nasal swabs were able to detect pertussis using a sensitive multitarget PCR. The less invasive mid-nasal nasal swab is an attractive alternative for pertussis nasal swab collection, and further research is needed to compare this collection site with nasopharyngeal swabs. In the future, this method may enhance population-based surveillance efforts.

## Supplementary Data

Supplementary materials are available at Journal of *The Pediatric Infectious Diseases Society* online.

## Notes

***Disclaimer.*** Neither of the funders had any role in the design and conduct of the study; collection, management, analysis, and interpretation of the data; or preparation, review, or approval of the manuscript.

***Financial support.*** This work was supported by grants from the Thrasher Research Fund (10470) and the Bill and Melinda Gates Foundation (50274).

***Potential conflicts of interest.*** J. A. E. has been a consultant for Pfizer, a member of a Data Safety Monitoring Board for GlaxoSmithKline (GSK) influenza antiviral studies, and her institution has received research support for clinical studies from GSK, Gilead, Chimerix, and Roche.

All authors have submitted the ICMJE Form for Disclosure of Potential Conflicts of Interest. Conflicts that the editors consider relevant to the content of the manuscript have been disclosed.

## Supplementary Material

Supplementary_Figure_1

Supplementary_Figure_2

eTable_1

eTable_2
