# Supplementary figures and images for: Population-Based Pertussis Incidence and Risk Factors in Infants Less Than 6 Months in Nepal
Source: J Pediatric Infect Dis Soc. 2017 Jan 10;6(1):33–9. doi: 10.1093/jpids/piw079 (PMC5907881; doi:10.1093/jpids/piw079)

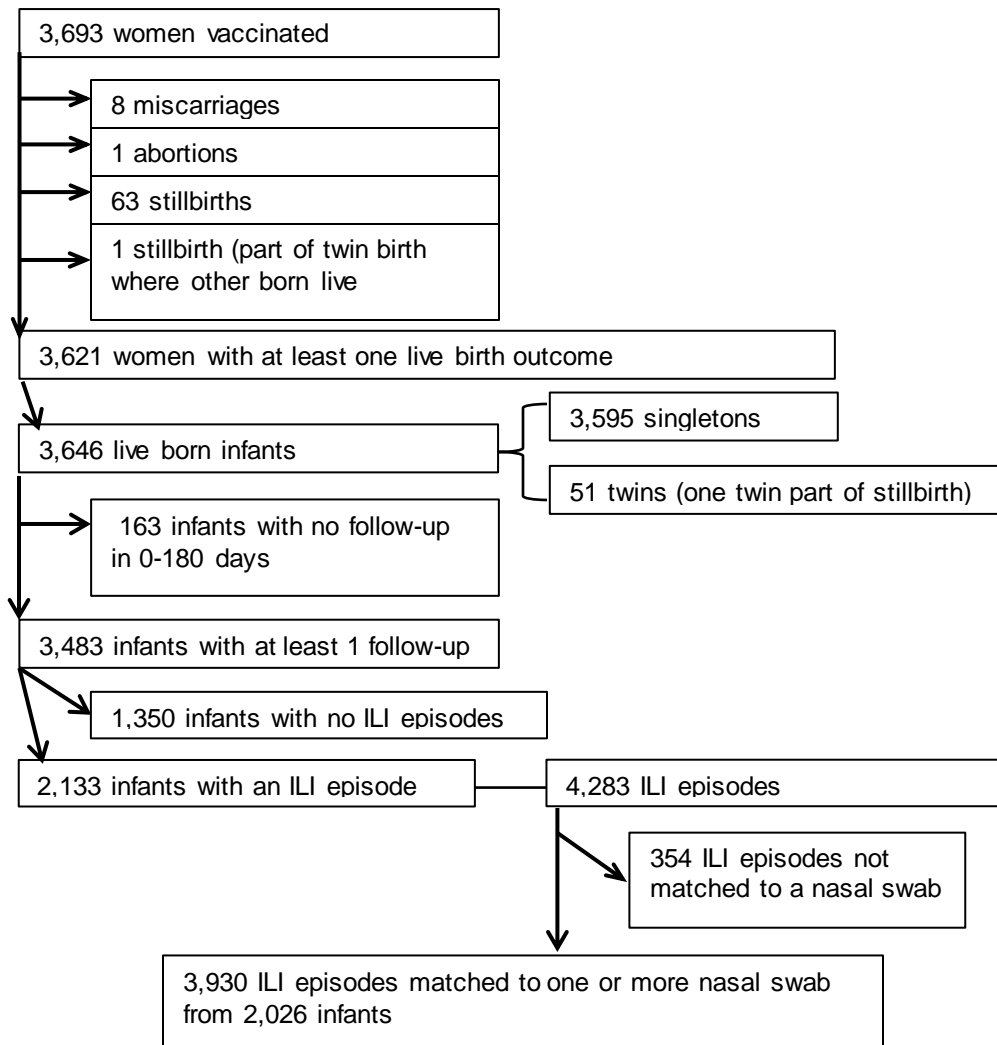

Supplementary Figure 1

Supplement: Supplementary_Figure_1 [file piw079_suppl_supplementary_figure_1.pdf]

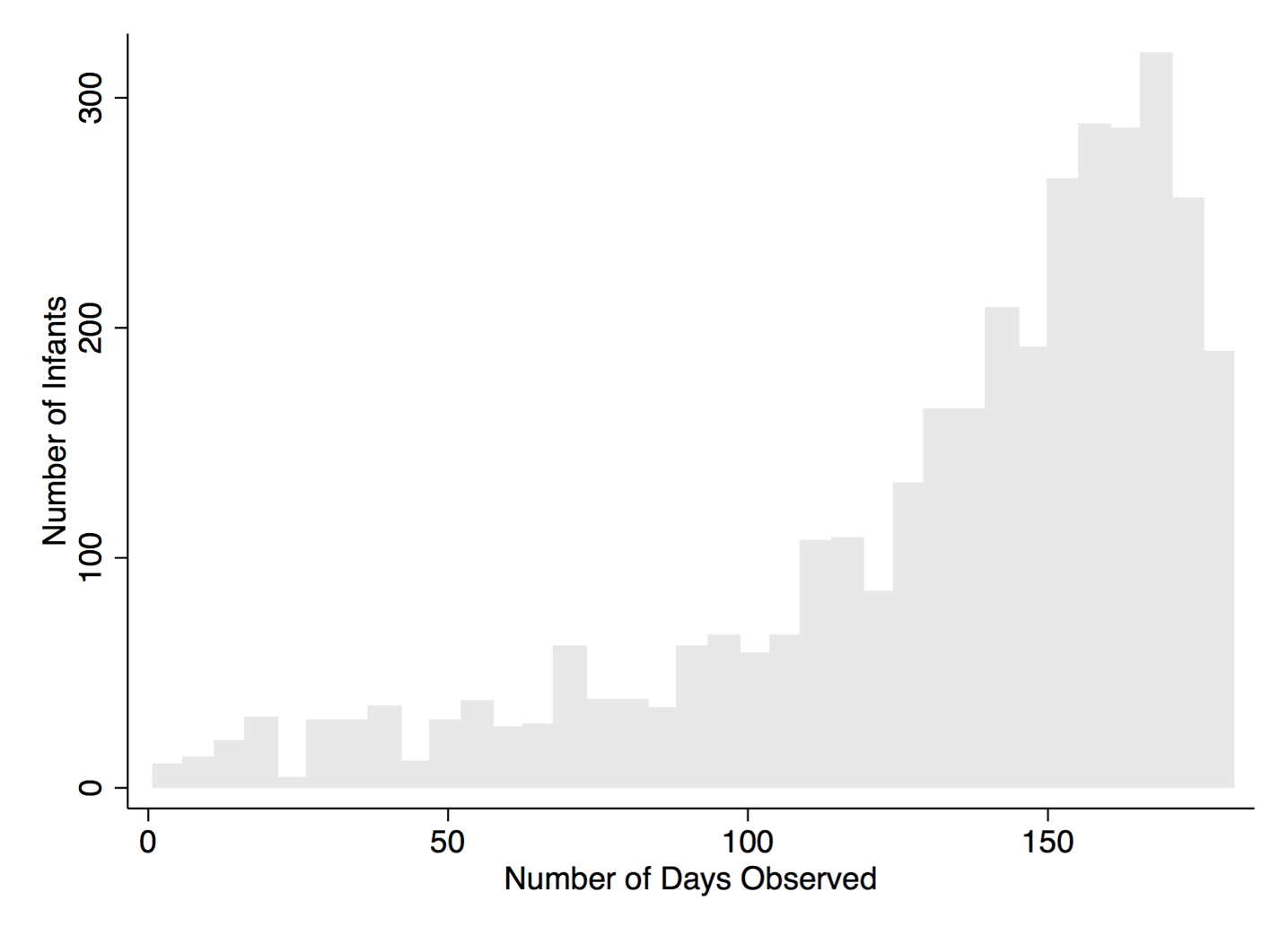


Supplementary Figure 2: Distribution of Number of Days of Infant Observation

Supplement: Supplementary_Figure_2 [file piw079_suppl_supplementary_figure_2.docx]
