# Supplementary material for: Population-Based Pertussis Incidence and Risk Factors in Infants Less Than 6 Months in Nepal
Source: J Pediatric Infect Dis Soc. 2017 Jan 10;6(1):33–9. doi: 10.1093/jpids/piw079 (PMC5907881; doi:10.1093/jpids/piw079)
Supplement: eTable_1 [file piw079_suppl_etable_1.docx]

**Supplementary Table 1**

| **Supplementary Table 1. Parapertussis Case Description** | | | | | |
| --- | --- | --- | --- | --- | --- |
|  |  |  |  |  |  |
| **Risk Factor** | **Case #1** | **Case #2** | **Case #3** | **Case #4** | **Case #5** |
|  |  |  |  |  |  |
|  |  |  |  |  |  |
| **Sex** | Male | Male | Male | Male | Female |
|  |  |  |  |  |  |
| **Preterm (<37 weeks)** | No | No | No | Preterm | No |
|  |  |  |  |  |  |
| **Low Birthweight (<2500 grams)** | No | No | No | - | No |
|  |  |  |  |  |  |
| **Small-for-Gestational Age (IG)^a^** | SGA | No | SGA | - | No |
| **Small-for-Gestational Age (A)^a^** | No | No | SGA | - | No |
|  |  |  |  |  |  |
| **Breastfed in 1st Hour** | Breastfed | No | No | Breastfed | No |
|  |  |  |  |  |  |
| **Parity** | non-primiparous | Primiparous | Primiparous | Primiparous | Primiparous |
|  |  |  |  |  |  |
| **Ethnicity** | Pahadi | Pahadi | Pahadi | Pahadi | Madeshi |
|  |  |  |  |  |  |
| **Literacy** | No | Literate | Literate | Literate | Literate |
|  |  |  |  |  |  |
| **Household size** | 5 | 9 | 10 | 3 | 1 |
|  |  |  |  |  |  |
| **SES Quartile^b^** | 2nd | 4th | 3rd | 2nd | 3rd |
|  |  |  |  |  |  |
| ^a^Small-for-gestational age: IG = INTERGROWTH-21st standards; A = Alexander standards | | | | |  |
| ^b^4th = highest SES |  |  |  |  |  |
