# Supplementary material for: Population-Based Pertussis Incidence and Risk Factors in Infants Less Than 6 Months in Nepal
Source: J Pediatric Infect Dis Soc. 2017 Jan 10;6(1):33–9. doi: 10.1093/jpids/piw079 (PMC5907881; doi:10.1093/jpids/piw079)
Supplement: eTable_2 [file piw079_suppl_etable_2.docx]

**Supplementary Table 2**

| **Supplementary Table 2. Parapertussis Episode Description** | | | | | | |
| --- | --- | --- | --- | --- | --- | --- |
|  |  |  |  |  |  |  |
| **Characteristic** | **Overall** | **Case #1** | **Case #2** | **Case #3** | **Case #4** | **Case #5** |
|  |  |  |  |  |  |  |
|  |  |  |  |  |  |  |
| **Symptoms** |  |  |  |  |  |  |
| Cough | 60% | Yes |  | Yes |  | Yes |
| Wheeze | 80% | Yes | Yes | Yes |  | Yes |
| Difficulty breathing | 20% |  |  |  | Yes |  |
| Fever | 20% | Yes |  |  |  |  |
| Ear Infection | 0% |  |  |  |  |  |
|  |  |  |  |  |  |  |
| **Episode duration (days)** | 4 | 6 | 2 | 5 | 3 | 4 |
| **Age at episode start (days)** | 57.8 | 67 | 7 | 95 | 49 | 71 |
|  |  |  |  |  |  |  |
| **Co-Infections^a^** |  |  |  |  |  |  |
| RV | 60% | Yes | Yes |  |  | Yes |
| RSV | 20% | Yes |  |  |  |  |
|  |  |  |  |  |  |  |
| ^a^No parapertussis cases were co-infected with AdV, BoV, Influenza, MPV, CoV, PIV1-4 | | | | | |  |
